# Identification and validation of key long non-coding RNAs in resveratrol protect against IL-1β-treated chondrocytes via integrated bioinformatic analysis

**DOI:** 10.1186/s13018-021-02574-4

**Published:** 2021-07-02

**Authors:** Hong Yi, Wei Zhang, Sheng-Yu Cui, Jian-Bo Fan, Xin-Hui Zhu, Wei Liu

**Affiliations:** Department of Orthopedic, the Affiliated Hospital 2 of Nantong Univeristy and the First People’s Hospital of Nantong, No. 6 Haierxiangbei Road, Nantong City, 226001 Jiangsu Province China

**Keywords:** Resveratrol, Osteoarthritis, LncRNAs, RNA sequencing

## Abstract

**Background:**

Long non-coding RNAs (lncRNAs) participate in regulation of gene transcription, but little is known about the correlation among resveratrol and lncRNAs. This study aimed to identify and validate the key lncRNAs in resveratrol protect against IL-1β-treated chondrocytes.

**Methods:**

In this experiment, high-throughput sequencing technique was performed to identify the differentially expressed lncRNAs, miRNAs, and mRNAs between IL-1β-treated chondrocytes with or not resveratrol. Moreover, gene ontology and KEGG pathway of the differentially expressed genes were carried out by R software. Then, lncRNA-miRNA-mRNA network was constructed by Cytoscape software. Venn diagram was performed to identify the potentially target miRNAs of LINC00654. Then, real-time polymerase chain reaction (RT-PCR) was performed to validate the most significantly differentially expressed lncRNAs.

**Results:**

Totally, 1016 differentially expressed lncRNAs were identified (493 downregulated) between control and resveratrol-treated chondrocytes. Totally, 75 differentially expressed miRNAs were identified (downregulated = 54, upregulated = 21). Totally, 3308 differentially expressed miRNAs were identified (downregulated = 1715, upregulated = 1593). GO (up) were as follows: skin development, response to organophosphorus. GO (down) mainly included visual perception, single fertilization, and sensory perception of smell. KEGG (up) were as follows: TNF signaling pathway and TGF-beta signaling pathway. KEGG (down) were as follows: viral protein interaction with cytokine and cytokine receptor. We identified that LINC00654 and OGFRL1 were upregulated in resveratrol-treated chondrocytes. However, miR-210-5p was downregulated in resveratrol-treated chondrocytes.

**Conclusion:**

In sum, the present study for the first time detected the differential expressed lncRNAs involved in resveratrol-treated chondrocytes via employing bioinformatic methods.

## Introduction

Osteoarthritis (OA) is a joint degenerative disease, which is common in middle-aged and elderly people [1, 2]. The pathological manifestations of OA are mainly including erosion of articular cartilage, marginal bone hyperplasia, and subchondral disease [3, 4]. It is reported that there are more than 2700 million OA patients in the USA [5, 6]. The prevalence rate of knee OA in people over 60 years old in China is as high as 49% [7].

OA is characterized by increases in the pro-inflammatory cytokines and apoptosis of chondrocytes [8]. Pathogenesis of OA is not yet fully understood and thus there is no effective treatment for OA [9]. Current treatment strategies focus on controlling pain symptoms and delay joint degeneration [10]. Therefore, there is an urgent need to develop new treatment options for OA.

Resveratrol (Res) is a non-flavonoid compound, which widely exist in the plant [11]. Studies have confirmed that there are as many as 21 species of plants are rich of Res, such as grapes, peanuts, and cassia [12]. Res was firstly isolated in the year of 1940 from the root of veratrumgrandiflorum. Large-scale experimental research found Res possess anti-aging, anti-oxidation, anti-inflammatory, anti-platelet aggregation, anti-atherosclerosis, anti-proliferation and anti-tumor activities, regulating cell apoptosis, and estrogen stimulation [13–15].

Long non-coding RNAs (lncRNAs) belong to a novel category of non-coding RNA molecules harboring over 200 nucleotides [16, 17]. Although lncRNAs exert no function of coding protein, they are extensively implicated in gene expression modulation, chromosome remodeling, and other physiological and pathological processing [18]. In recent years, the expression profile and detailed functions of lncRNAs in osteoarthritis have received more and more interest and are being heavily studied [19].

The aberrant expression of lncRNAs is frequently identified in osteoarthritis, implying that they may take part in the progression of osteoarthritis. An accumulating number of evidence has illuminated the anti-inflammation activity of lncRNAs, thus exerting important regulatory roles in osteoarthritis [20].

MicroRNAs (miRNAs) are about 17–21 nucleotides in length and are defined as a group of single-strand non-coding RNA transcripts [21, 22]. They post-transcriptionally control genes expression through complementary interacting with their target genes, consequently blocking the translations and protein accumulation [23]. LncRNAs and miRNAs can interact with each other, thus affecting the metabolic activities of cells. In the fields of competitive endogenous RNA (ceRNA) theory, lncRNAs harbor miRNA response elements and can competitively bind to miRNAs, consequently weakening the repressive effect of miRNAs on target mRNAs and regulating genes at posttranscriptional level [24]. Therefore, the exploration of lncRNAs in osteoarthritis may be vital for the discovery of novel diagnostic and therapeutic targets.

In this study, we performed RNA sequencing about control and resveratrol-treated chondrocytes and then construct lncRNA-miRNA-mRNA network. Therefore, the present study may advance the understanding of the underlying molecular mechanisms of OA and may contribute to the diagnosis and treatment of OA.

## Material and methods

### Chondrocytes isolated and cultured

Chondrocytes were isolated as previously described [25]. The cartilage tissues from patients that underwent total knee arthroplasty were cut into 3–5 mm^3^ slices. Then, the slices were digested with 0.2% collagenase type II to isolate primary chondrocytes. Then, chondrocytes were washed with PBS for three times and then incubated in a humidified incubator (37 °C and 5% CO_2_) for 24 h. To mimic the OA inflammatory condition, IL-1β (10 ng/ml, 2 h) was chosen to treat human chondrocytes directly. To study the treatment of resveratrol, the chondrocytes were prematurely exposed to 25 μM resveratrol and then treated with IL-1β (10 ng/ml, 2 h).

### RNA sequencing

RNA from chondrocytes treated with IL-1β or IL-1β + resveratrol was extracted with TRIzol by standard methods. Concentration of total RNA, RIN value, 28S/18S, and fragment size were detected with Agilent 2100 Bioanalyzer (Agilent RNA 6000 Nano Kit). Agarose gel electrophoresis was used to detect RNA integrity. The cDNA library was established using the RNA-Seq Library Preparation Kit (Illumina) following the manufacturer’s protocol. Sequencing was performed on the Illumina HiSeq4000 sequencing platforms at Majorbio Biotech Co., Ltd.

### Bioinformatic analysis

Normalization was performed using median normalization. Differentially expressed genes (DEGs) analysis was performed with R software, using package DESeq2. Heatmap and volcano plot visualizations were performed using the R package “pheatmap” and “ggplot2,” respectively. Gene Ontology (GO) and KEGG pathway enrichment were performed using DAVID software (https://david-d.ncifcrf.gov/). lncRNA–miRNA and miRNA–mRNA pairs were used to construct a lncRNA–miRNA–mRNA network using Cytoscape v.3.6.1 software. The binding interaction between LINC00654 and miR-210-5p was predicted utilizing StarBase 3.0 (http://starbase.sysu.edu.cn/). Three bioinformatics tools, namely TargetScan (http://www.targetscan.org/), miRDB (http://mirdb.org/), and StarBase 3.0, were used to forecast the possible target genes of miR-210-5p.

### RNA preparations and quantitative reverse transcription–polymerase chain reaction (qRT-PCR)

The extraction of total RNA from collected samples or cells was carried out utilizing total RNA Purification Kit (Norgen Biotek Corp., Belmont, CA, USA). RNA was subjected to a NanoDrop™ 2000 Spectrophotometer (Invitrogen; Thermo Fisher Scientific, Inc.) for the determination of RNA purity and concentration. The first-strand complementary DNA was prepared from total RNA with a PrimeScript™ RT Reagent kit with gDNA Eraser (Takara Biotechnology Co., Tokyo, Japan). Then, PCR amplification was implemented with a TB Green Premix Ex Taq (Takara Biotechnology Co.). Glyceraldehyde-3-phosphate dehydrogenase (GAPDH) was taken as the control for target gene expression. U6 small nuclear RNA served as the internal reference for miRNA. All genes expression was analyzed by 2^−ΔΔCt^ method.

### Dual luciferase reporter assay

LINC00654 or OGFRL1 containing the putative binding sites of miR-210-5p was obtained from Genepharma and cloned into the vectors (pGL6; Beyotime) for establishment of reporter vectors LINC00654 (WT/MT) or OGFRL1 (WT/MT). LINC00654 (WT/MT) or OGFRL1 (WT/MT) was transfected into chondrocytes together with vector-control (NC) or miR-210-5p mimic using Lipofectamine 2000 (Thermo Fisher Scientific). The result was analyzed by the Dual-Glo Luciferase Assay System (Promega).

### Apoptosis

Briefly, chondrocytes (3 × 10^5^/ml) were seeded in a 6-well plate and incubated for 24 h. The cells were then incubated with IL-1β (10 ng/ml) at 37 °C. Cell suspension was collected in 15 mL centrifugation and centrifuge at 1000 rpm for 5 min in the tube. Then, 1 mL PBS was added to resuspend the cells and then centrifuge again at 1000 rpm for 5 min. Five microliters of Annexin V-FITC was added and incubated at room temperature in the dark for 10 min. Five microliters of PI was then incubated at room temperature for 5 min in the dark. Cell apoptosis was examined by a flow cytometer (BD Biosciences) and then was analyzed using Flowjo version 7.6 software (Flowjo LLC).

### Statistical analysis

Nine biological replicates were performed for each experiment and mean values were calculated. Differences among multiple cell transfection groups were analyzed using ANOVA (one-way) and Tukey’s test. P < 0.05 was statistically significant.

## Results

### Identify DE-LncRNA between Res and normal chondrocytes

**Fig. 1:**
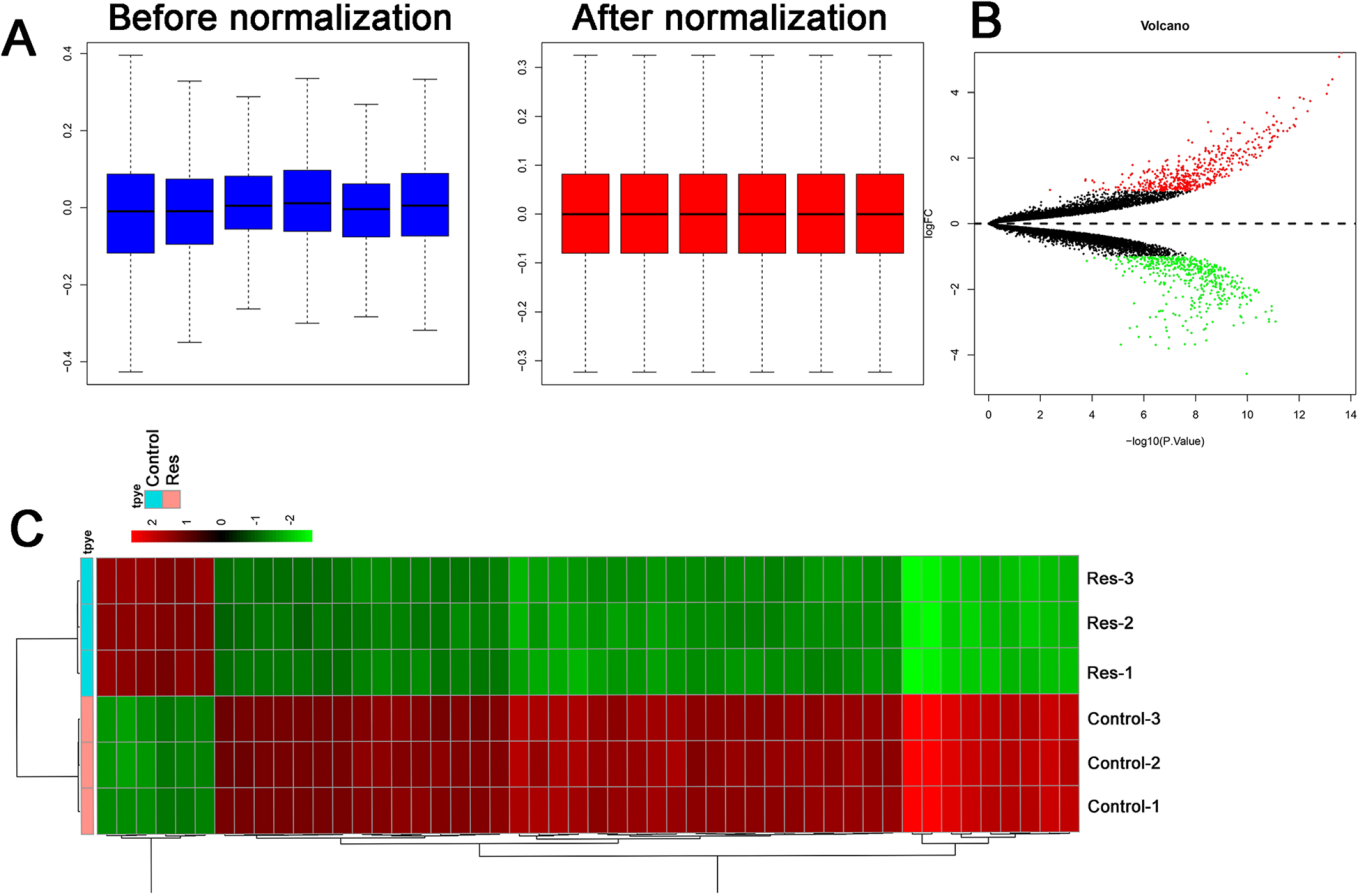
Differentially expressed lncRNA in resveratrol-treated chondrocytes. **A** Comparison of expression value between before normalization and after normalization; the vertical axis represents the different treatment samples, and the horizontal axis represents gene expression level (log2 scale). **B** Volcano plot of the differentially expressed lncRNAs in resveratrol-treated chondrocytes, red dots represented upregulated genes, green dots represented downregulated genes, and black dots represented non-significant genes. **C** Heatmap of the differentially expressed lncRNAs in resveratrol-treated chondrocytes; the color (from green to red) represents gene expression intensity from low to high

After normalization, Fig. 1A depicts that the log 2 ratios in the six pairs of samples are almost identical. Totally, 1016 differentially expressed lncRNAs were identified (493 downregulated, 523 upregulated). The expression heatmap and volcano plot are presented in Fig. 1B, C respectively.

### Identify DE-miRNAs between Res and normal chondrocytes

**Fig. 2:**
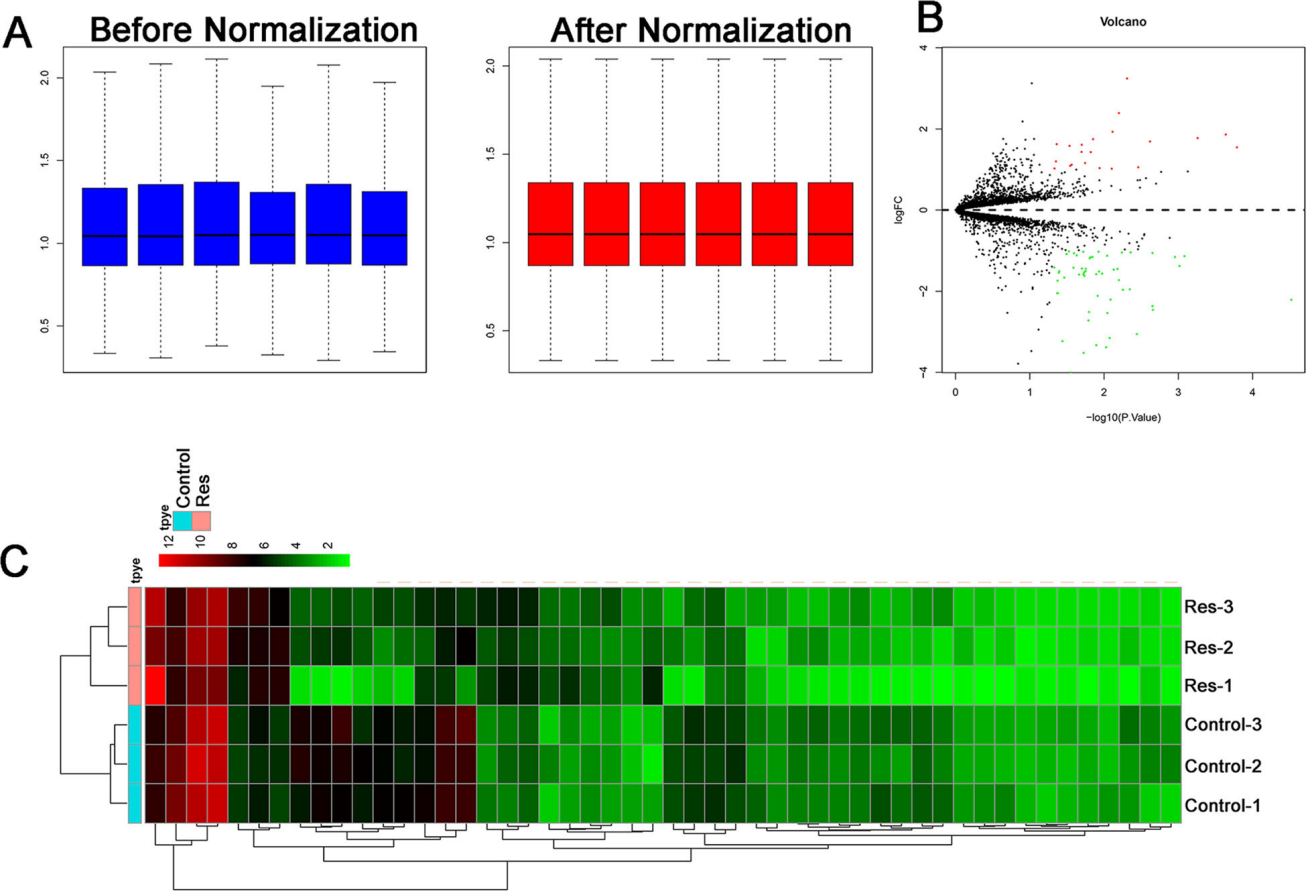
Differentially expressed miRNAs in resveratrol-treated chondrocytes. **A** Comparison of expression value between before normalization and after normalization. **B** Volcano plot of the differentially expressed miRNAs in resveratrol-treated chondrocytes. **C** Heatmap of the differentially expressed miRNAs in resveratrol-treated chondrocytes

Expression data was normalized and the log 2 ratios in the six pairs of samples are almost identical after normalization (Fig. 2A). Totally, 75 differentially expressed miRNAs were identified (downregulated = 54, upregulated = 21). The expression heatmap and volcano plot are presented in Fig. 2B, C respectively.

### Identify DE-mRNAs between Res and normal chondrocytes

**Fig. 3:**
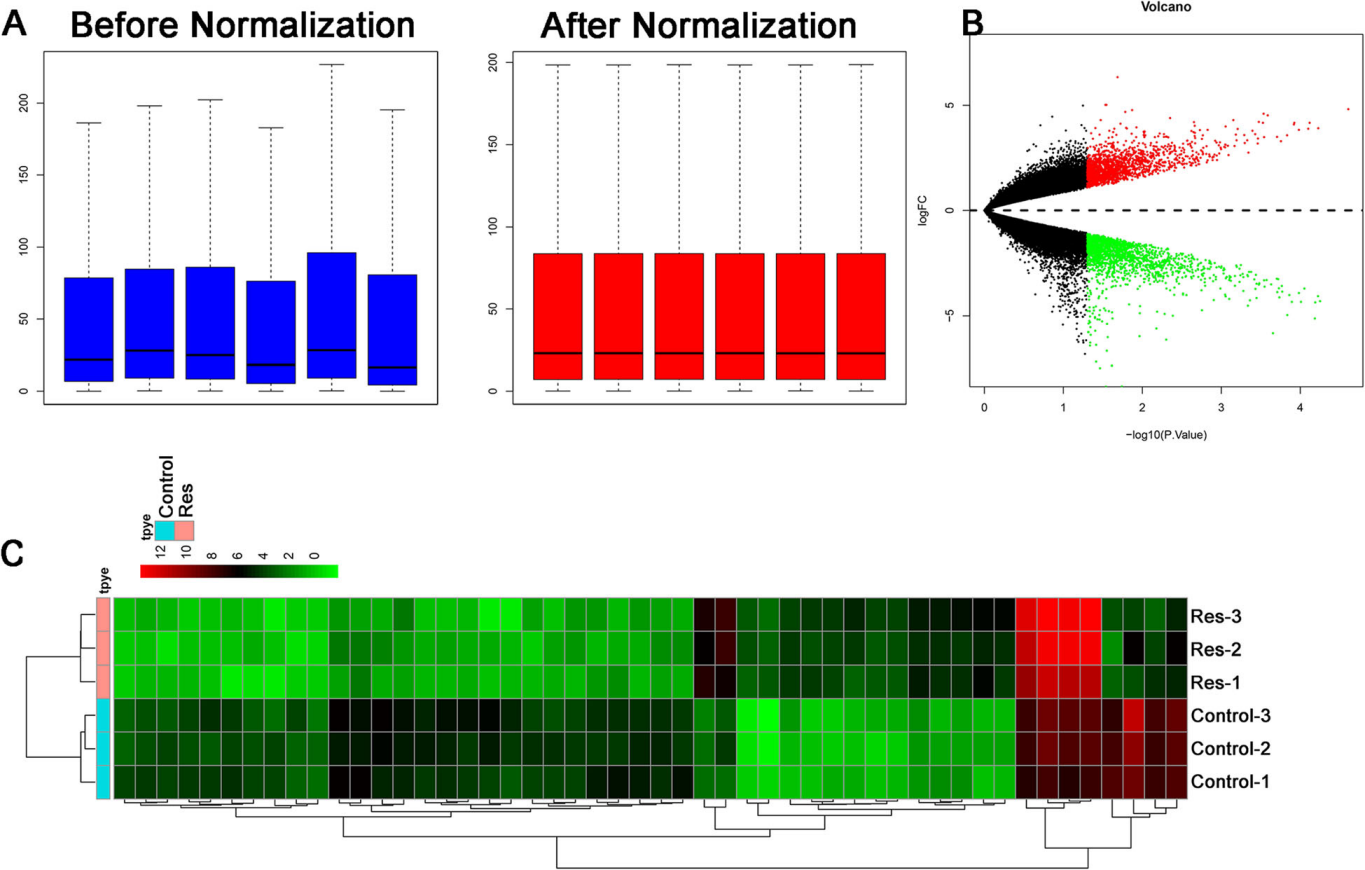
Differentially expressed mRNAs in resveratrol-treated chondrocytes. **A** Comparison of expression value between before normalization and after normalization. **B** Volcano plot of the differentially expressed mRNAs in resveratrol-treated chondrocytes. **C** Heatmap of the differentially expressed mRNAs in resveratrol-treated chondrocytes

After normalization, the log 2 ratios in the six pairs of samples are almost identical, which indicated that these data could be used for further analysis (Fig. 3A). Totally, 3308 differentially expressed miRNAs were identified (downregulated = 1715, upregulated = 1593). The expression heatmap and volcano plot are presented in Fig. 3B, C respectively.

### Gene ontology of the differentially expressed genes

**Fig. 4:**
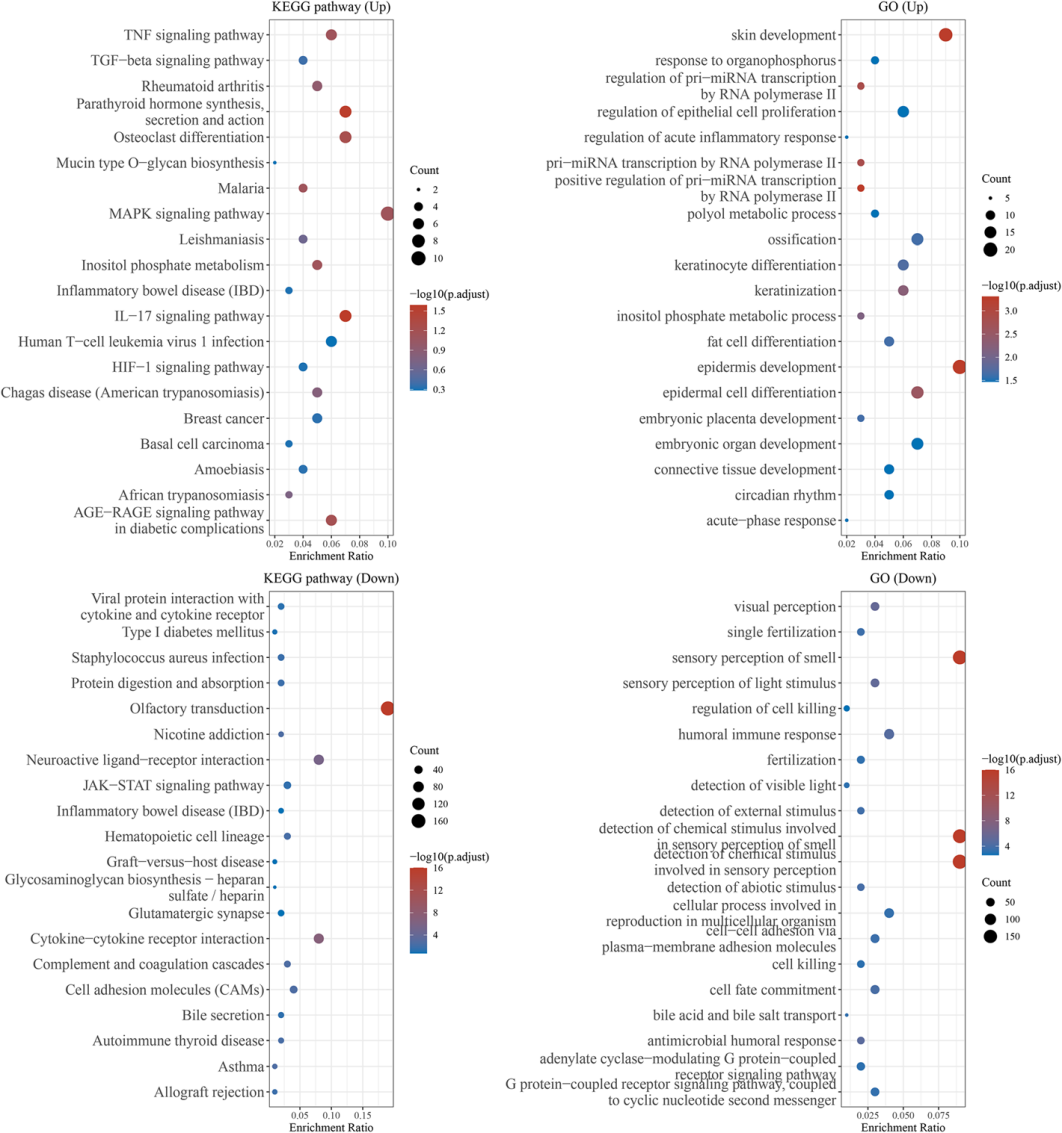
Gene ontology and KEGG pathway analysis of the differentially expressed genes in resveratrol-treated chondrocytes

The differentially expressed genes were categorized using Gene Ontology (GO). We divided into GO (up) and GO (down). GO (up) included skin development, response to organophosphorus, regulation of pri-miRNA transcription by RNA polymerase II, regulation of epithelial cell proliferation, regulation of acute inflammatory response, pri-miRNA transcription by RNA polymerase II, positive regulation of pri-miRNA transcription by RNA polymerase II, polyol metabolic process, ossification, keratinocyte differentiation, keratinization, inositol phosphate metabolic process, fat cell differentiation, epidermis development, epidermal cell differentiation, embryonic placenta development, embryonic organ development, connective tissue development, circadian rhythm, and acute-phase response (Fig. 4).

GO (down) included visual perception, single fertilization, sensory perception of smell, sensory perception of light stimulus, regulation of cell killing, humoral immune response, fertilization, detection of visible light, detection of external stimulus, detection of external stimulus detection of chemical stimulus involved in sensory perception of smell, detection of chemical stimulus involved in sensory perception of smell, detection of abiotic stimulus, cellular process involved in reproduction in multicellular organism cell-cell adhesion via plasma-membrane adhesion molecules, cell killing, cell fate commitment, bile acid and bile salt transport, antimicrobial humoral response, adenylate cyclase-modulation G protein-coupled receptor signaling pathway, and G-protein-coupled receptor signaling pathway, coupled to cyclic nucleotide second messenger (Fig. 4).

KEGG (up) were as follows: TNF signaling pathway, TGF-beta signaling pathway, rheumatoid arthritis, parathyroid hormone synthesis, secretion and action, osteoclast differentiation, mucin type O-glycan biosynthesis, malaria, MAPK signaling pathway, leishmaniasis, inositol phosphate metabolism, inflammatory bowel disease, IL-17 signaling pathway, human T cell leukemia virus 1 infection, HIF-1 signaling pathway, Chagas disease, breast cancer, basal cell carcinoma, amoebiasis, African trypanosomiasis, and AGE-RAGE signaling pathway in diabetic complications (Fig. 4).

KEGG (down) were as follows: viral protein interaction with cytokine and cytokine receptor, type I diabetes mellitus, Staphylococcus aureus mellitus, Staphylococcus aureus infection, protein digestion and absorption, olfactory transduction, nicotine addiction, neuroactive ligand-receptor interaction, JAK-STAT signaling pathway, inflammatory bowel disease, Hematopoietic cell lineage, graft-versus-host disease, glycosaminoglycan biosynthesis-heparan sulfate/heparin, glutamatergic synapse, cytokine-cytokine receptor interaction, complement and coagulation cascades, cell adhesion molecules, bile secretion, autoimmune thyroid disease, asthma, and allograft rejection (Fig. 4).

### Protein–protein interaction

**Fig. 5:**
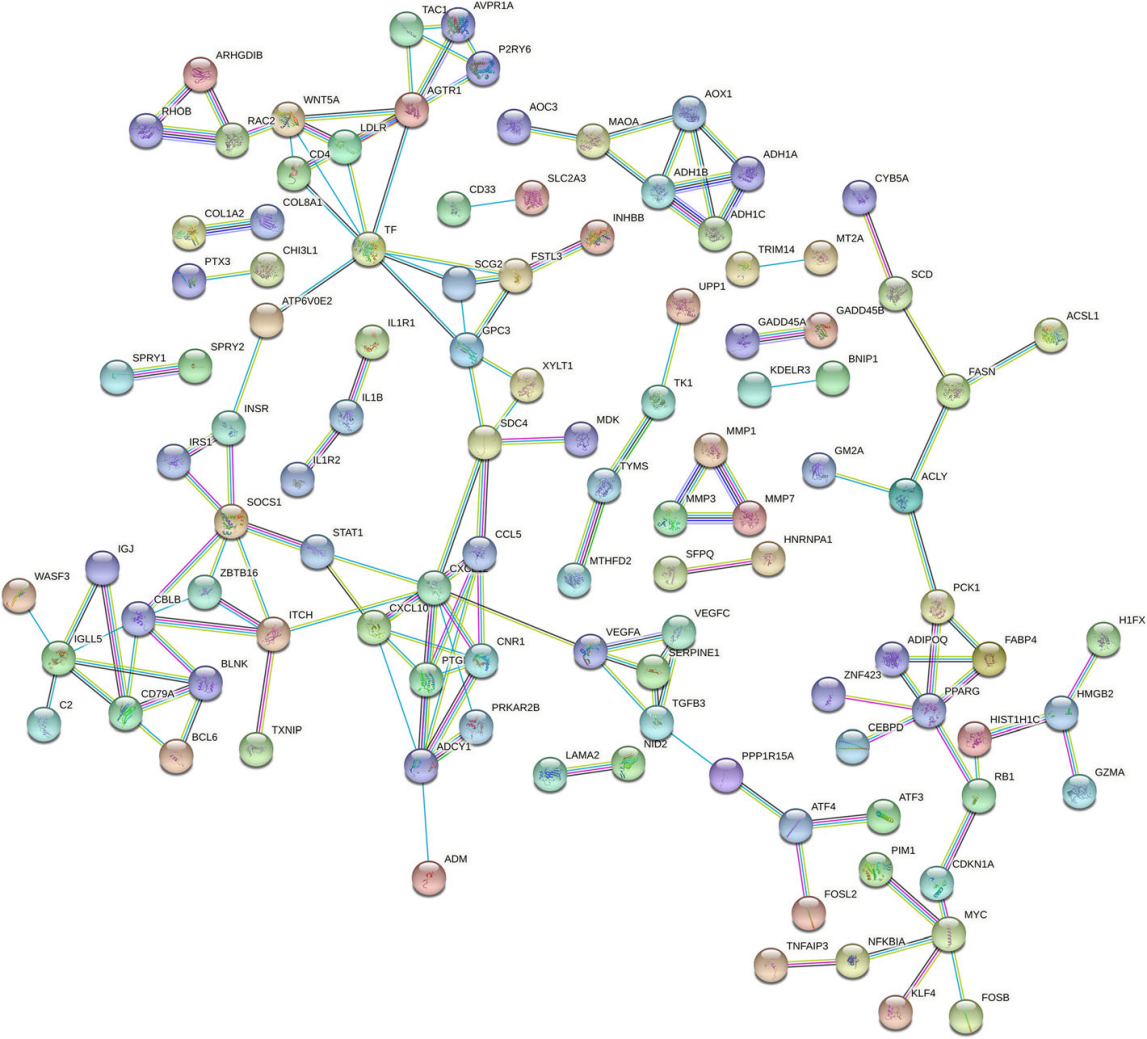
Protein-protein interaction of the differentially expressed genes in resveratrol-treated chondrocytes

Protein–protein interaction networks were constructed using STRING database (https://www.string-db.org/). A total of 311 DEGs were imported into the PPI network complex of 250 nodes and 141 edges, including 303 upregulated and 87 downregulated genes (Fig. 5).

### ceRNA network construction

**Fig. 6:**
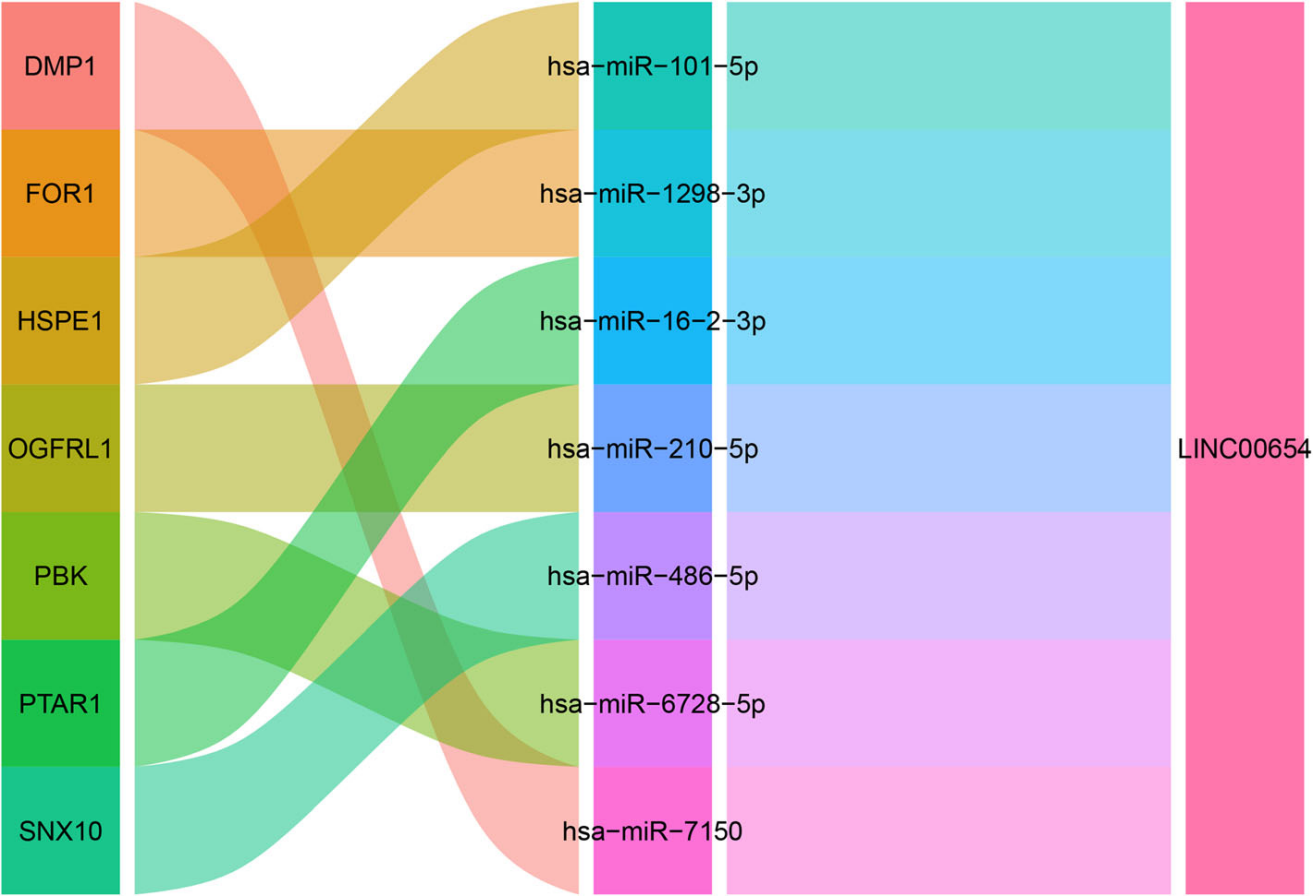
CeRNA network of LINC00654 and target miRNAs

The differentially expressed lncRNAs between the IL-1β-treated chondrocytes and the Res-treated chondrocytes were obtained (Fig. 6).

### Identify the LINC00654, miR-210-5p, and OGFRL1 expression

**Fig. 7:**
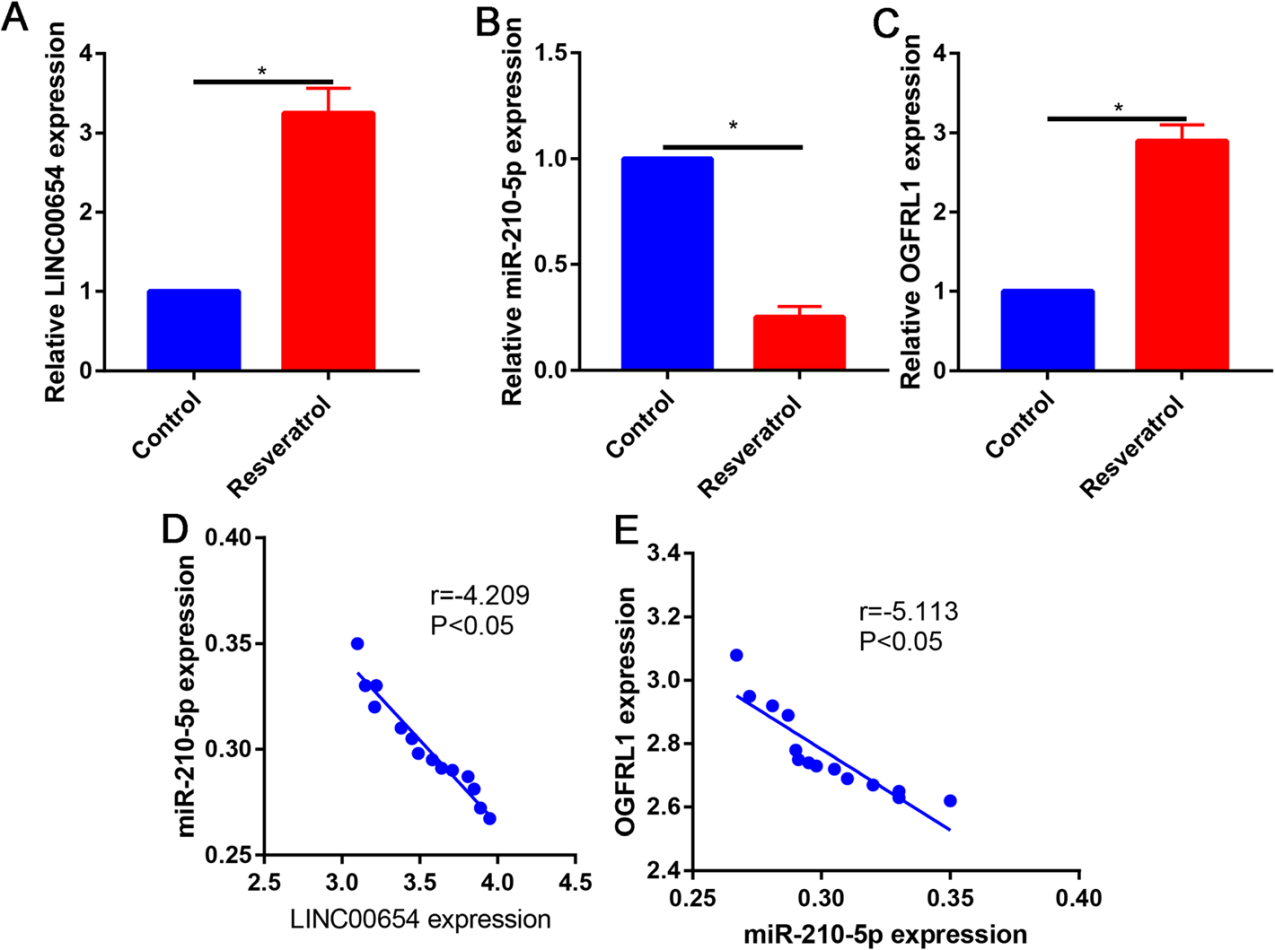
Relative LINC00654 expression in control and resveratrol-treated chondrocytes. **B** Relative miR-210-5p expression in control and resveratrol-treated chondrocytes. **C** Relative OGFRL1 expression in control and resveratrol-treated chondrocytes. **D** Correlation between LINC00654 and miR-210-5p. **E** Correlation between miR-210-5p and OGFRL1. *P < 0.05

Compared with control group, the LINC00654 expression was significantly upregulated in resveratrol-treated chondrocytes (Fig. 7A). Compared with control group, the miR-210-5p expression was significantly downregulated in resveratrol-treated chondrocytes (Fig. 7B). Compared with control group, the OGFRL1 expression was significantly upregulated in resveratrol-treated chondrocytes (Fig. 7C). There was a negative correlation between LINC00654 and miR-210-5p in resveratrol-treated chondrocytes (Fig. 7D). There was a negative correlation between miR-210-5p and OGFRL1 in resveratrol-treated chondrocytes (Fig. 7E).

### LINC00654 directly target with miR-210-5p

**Fig. 8:**
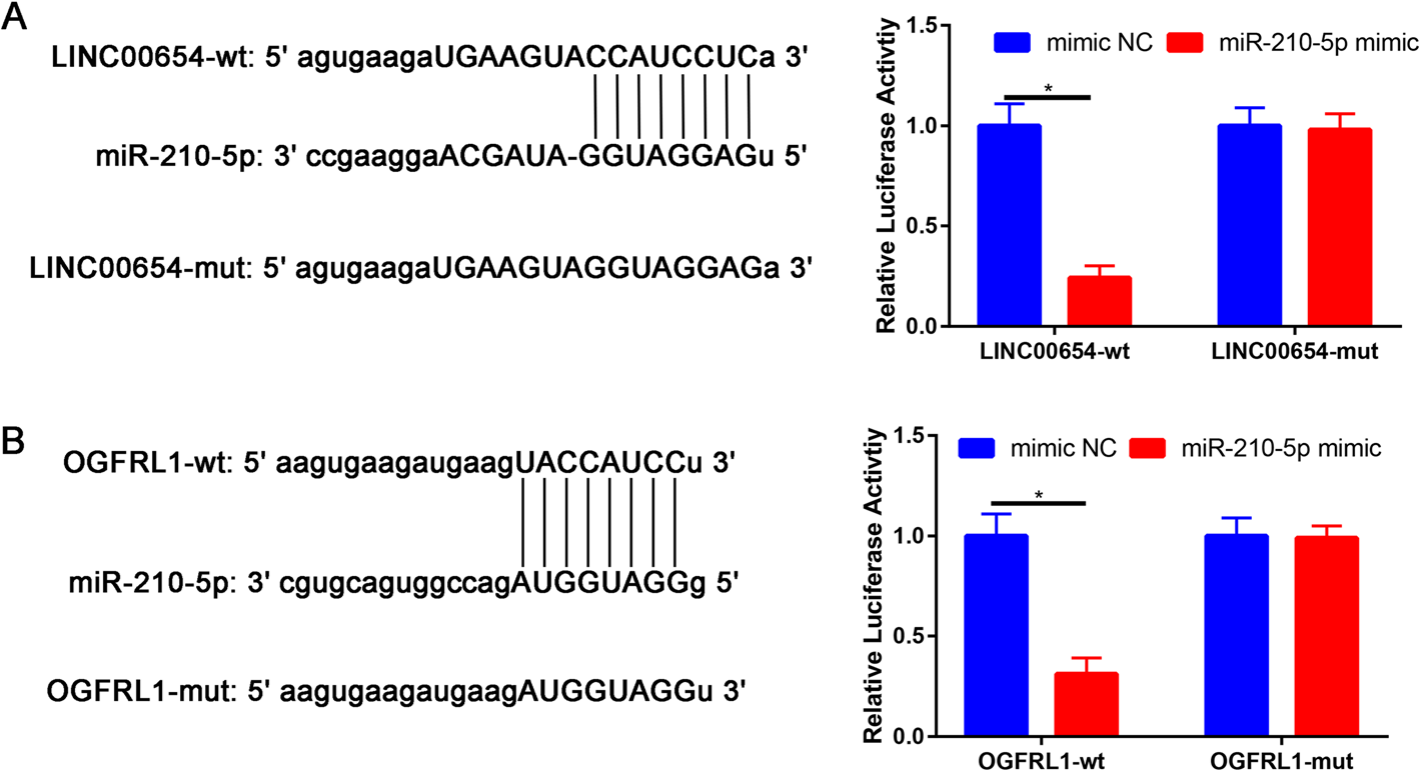
**A** The gene structure of LINC00654 at positions 1432-1444 contained the predicted target site of miR-210-5p in its 3′-UTR. Luciferase activity was measured with a dual luciferase reporter assay in chondrocytes co-transfected with the WT/MT LINC00654 3′-UTR plasmid and miR-210-5p. **B** Luciferase activity was measured with a dual luciferase reporter assay in chondrocytes co-transfected with the WT/MT OGFRL1 3′-UTR plasmid and miR-210-5p

In addition, the relative luciferase activity in WT-LINC00654 or WT-OGFRL1 was significantly inhibited by miR-210-5p mimic (Fig. 8A). However, miR-210-5p mimic did not affect the luciferase activity in WT-LINC00654 or WT-OGFRL1 (Fig. 8B).

### Cell apoptosis

**Fig. 9:**
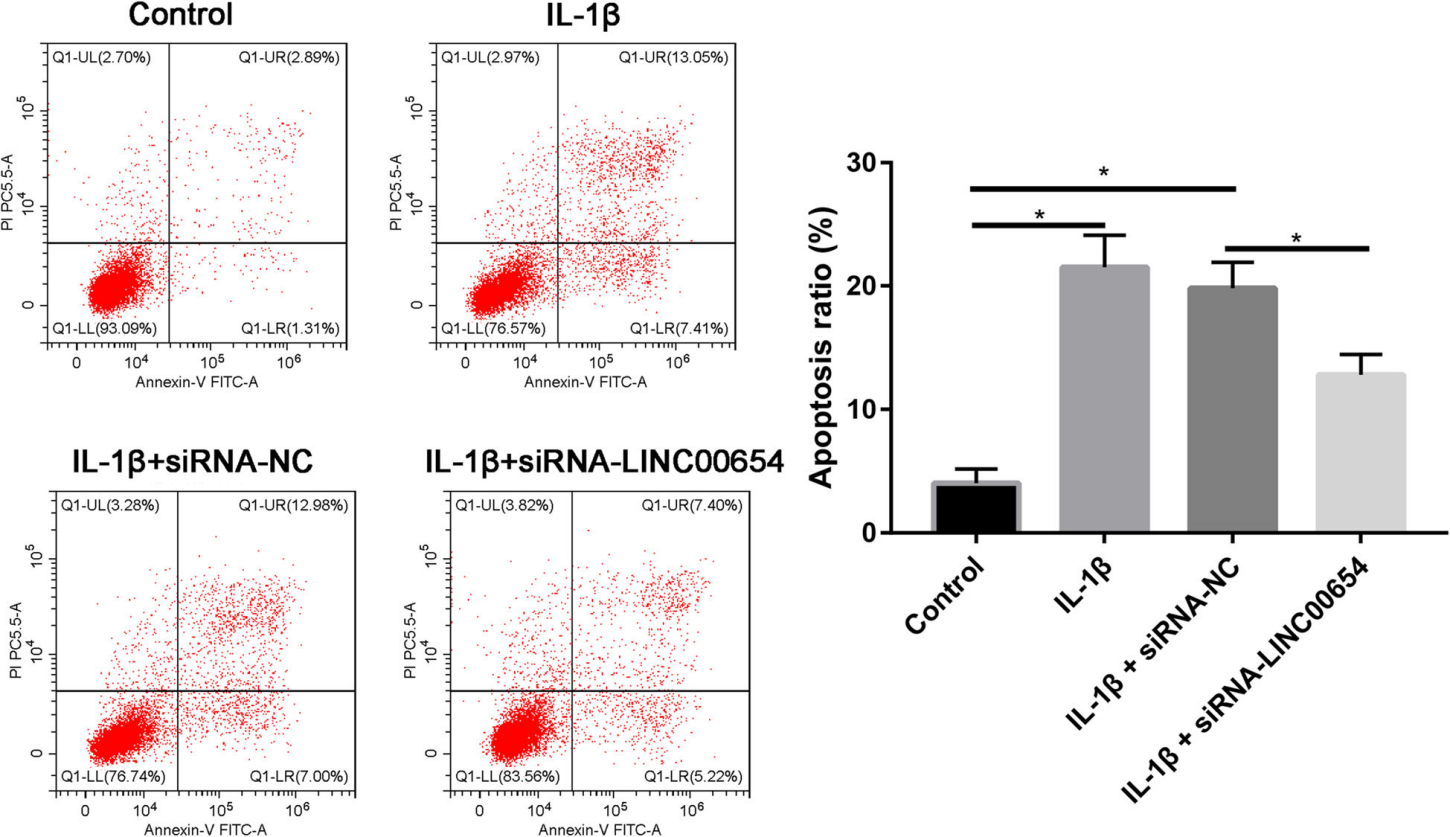
OA articular chondrocytes were either transfected with non-specific control (control) without (IL−) or with IL-1β stimulation (IL+) for 12 h, or transfected with si LINC00654 with IL-1β stimulation (IL+) for 12 h. *P < 0.05

Cell apoptosis was analyzed by Annexin V assays followed by flow cytometry. IL-1β significantly increased the apoptotic ratio than control group, while these pro-apoptotic effects were reversed by silencing LINC00654 (Fig. 9).

## Discussion

This is the first study that aimed to identify the differentially expressed lncRNAs, miRNAs, and mRNAs between resveratrol and control. High-throughput RNA sequencing was performed and we selected one lncRNA to validate. We construct lncRNA-miRNA-mRNA network to explain the mechanism of resveratrol in protecting against osteoarthritis. Previous study has identified that resveratrol alleviates the IL-1β-induced chondrocytes injury through the NF-κB signaling pathway [26]. Xu et al. [27] revealed that resveratrol inhibits the development of obesity-related osteoarthritis via the TLR4 and PI3K/Akt signaling pathways. Further studies confirmed their findings and identified that resveratrol alleviating obesity-related osteoarthritis via alleviating JAK2/STAT3 signaling pathway, which independent of SOCS3. Zhang et al. [28] found that resveratrol could prevent osteoarthritis progression via the MALAT1/miR-9/NF-κB signaling pathway. Further in vivo study found that resveratrol ameliorates inflammatory damage and protects against osteoarthritis in a rat model of osteoarthritis [29]. However, there is no study about the resveratrol for regulating lncRNA-miRNA-mRNA regulation network.

With the improvement of high-throughput sequencing, more lncRNAs were identified. A total of 1016 differentially expressed lncRNAs were identified between resveratrol-treated chondrocytes.

A substantial body of evidences have implicated that lncRNAs participated into the osteoarthritis progression. Luo et al. [30] found that knockdown of lncRNA MFI2-AS1 inhibits lipopolysaccharide-induced osteoarthritis progression by miR-130a-3p/TCF4. Huang et al. [31] revealed that lncRNA DILC is downregulated in osteoarthritis and regulates IL-6 expression in chondrocytes. Tang et al. [32] found that lncRNA TUG1 promotes osteoarthritis-induced degradation of chondrocyte extracellular matrix via miR-195/MMP-13 axis. In this study, we further performed RT-PCR and identify the expression of LINC00654 in control and resveratrol-treated chondrocytes. Further study identified that LINC00654 has putative targeting sites with miR-210-5p through bioinformatic analysis. Wu et al. [33] performed a miRNA sequencing and identified miR-210-5p is highly enriched in OA sclerotic subchondral bone osteoblast exosomes. Gu et al. [34] found that oral resveratrol prevents osteoarthritis progression in C57BL/6J mice fed a high-fat diet. These researches verified that resveratrol could be a potential therapeutic target for osteoarthritis.

## Conclusion

In sum, the present study for the first time detected the differential expressed lncRNAs involved in resveratrol-treated chondrocytes via employing bioinformatic methods. An lncRNA-associated ceRNA network was established and hub lncRNA, such as LINC00654, were identified. Functional enrichments disclosed that resveratrol could regulate LINC00654 expression, which could compete with OGFRL1 to combine with miR-210-5p.

## Data Availability

We state that the data will not be shared since all the raw data are present in the figures included in the article.

## Abbreviations

lncRNAs: Long non-coding RNAs
PCR-RT: Polymerase chain reaction–real-time
OA: Osteoarthritis
Res: Resveratrol
miRNAs: MicroRNAs
ceRNA: Competitive endogenous RNA
DEGs: Differentially expressed genes
